# Hot-Pressing Process of Flat-Pressed Wood–Polymer Composites: Theory and Experiment

**DOI:** 10.3390/polym16202931

**Published:** 2024-10-18

**Authors:** Pavlo Lyutyy, Pavlo Bekhta, Yurii Protsyk, Vladimír Gryc

**Affiliations:** 1 Department of Wood-Based Composites, Cellulose and Paper, Ukrainian National Forestry University, 79057 Lviv, Ukraine; lyutyj_p@nltu.edu.ua (P.L.); protsyk@nltu.edu.ua (Y.P.); 2 Green Cotton Group A/S, 7430 Ikast, Denmark; 3 Department of Wood Science and Technology, Mendel University in Brno, 613 00 Brno, Czech Republic; vladimir.gryc@mendelu.cz; 4 Department of Furniture and Wood Products, Technical University in Zvolen, 960 01 Zvolen, Slovakia

**Keywords:** wood–polymer composites, flat pressing, pressing time, pressing temperature

## Abstract

The objective of this research was to develop a mathematical model of the hot-pressing process for making flat-pressed wood–polymer composites (FPWPCs). This model was used to calculate and predict the temperature and time required for FPWPC pressing. The model’s performance was analysed using the experimental results of hot pressing FPWPCs. It was found that an increase in the content of wood particles led to a rapid increase in the pressing time. The model and experiment showed that the core temperature of the wood–polymer mat remained nearly constant for the first 20–30 s of the hot-pressing process. After this period, this temperature increased rapidly until it reached 100 °C, after which the rate of increase began to decelerate sharply. This transition was more distinct in FPWPCs with a high wood-particle content, while in those with a high thermoplastic-polymer content, it was smoother. Increasing the pressing temperature contributed to a reduction in the time required to heat the FPWPC, as confirmed by both experimental data and the modelling of the hot-pressing process. A decrease in the predicted density of the FPWPC resulted in a directly proportional increase in the time required to heat the mat. Validation of the mathematical model revealed a mean absolute percentage error (MAPE) of only 2.5%, confirming its high precision and reliability. The developed mathematical model exhibited a high degree of accuracy and can be used for further calculations of the time required for FPWPC pressing, considering variable parameters such as pressing temperature, wood–polymer ratio, mat thickness, and density.

## 1. Introduction

Wood–polymer composites (WPCs) have gained significant commercial importance in recent years. They offer numerous advantages over conventional wood composites made from thermosetting resins and mineral binders, including high water resistance, non-toxicity, chemical resistance, and recyclability [1,2]. WPCs are usually made by the extrusion method, which makes it possible to produce products of unlimited length with a small cross-section. However, this method is unsuitable for manufacturing large, flat composite panels [1,2]. Flat pressing is a promising alternative, though this method is still underdeveloped for WPC production and requires further research [3,4,5]. To achieve high strength and water resistance in flat-pressed WPCs (FPWPCs), the core layer must be heated to a temperature high enough to melt the thermoplastic polymer but not so high as to cause the degradation of the polymer or the wood components [5]. The degradation of large-molecule wood components actually begins at temperatures slightly above 100 °C, though it remains negligible up to 150 °C [6]. Hemicelluloses in wood begin to degrade at a significant rate at temperatures exceeding 150 °C. The most intense degradation is observed in the range of 200–270 °C; the maximum rate is observed at 240–245 °C, where approximately 50% of the hemicellulose mass is converted into volatile and gaseous products. In consequence, pressing wood composites at temperatures above 200 °C is not recommended, as the volatile and gaseous substances formed from the degradation of the wood components increase internal stresses in the material, forming cracks and unbonded areas and significantly degrading the physical and mechanical properties of the composite [6]. Thus, the properties of the final product are mainly determined by the pressing process, since this involves the consolidation and bonding of the material’s components [7].

Many researchers have investigated the process of pressing wood composite materials. Some have proposed mathematical models of the process for particleboard [8,9], medium-density fibreboard (MDF) [10,11,12,13], and oriented strand board (OSB) [14,15]. These mathematical models make it possible to establish the influence of numerous factors on the pressing process of wood composites. Specifically, one study [9] determined the core temperature of particleboards during pressing and investigated the influence of parameters such as material moisture content, pressing temperature, and material type. Mathematical models have been developed of both batch- and continuous-pressing processes for wood composite materials [16,17]. A comparative study of the empirical and fundamental models of hot pressing MDF has also been made [18]. However, the applicability to WPCs of the existing mathematical models of particleboard, MDF, and OSB is limited because of the significant differences in their structure and process conditions. This means that the accuracy of such models in predicting the properties of WPCs will be low.

Thermoplastic polymers have different thermal characteristics from thermosetting resins. Thermoplastics undergo a softening process when exposed to heat below their melting point and exhibit fluid-like behaviour under pressure above this temperature [1,2]. Their heat capacity and conductivity vary with temperature, exhibiting different trends below and above their melting point. It has been found that the core temperature of the FPWPC mat during manufacture significantly affects the physical and mechanical properties of the resulting panel. The properties of FPWPCs improve with increasing core mat temperature; however, enhancing these properties may not justify the extra cost of using more heat in processing [19].

Models have been developed to investigate the influence of various factors on the mechanical [20,21] and thermal [21] properties of WPCs. However, these models apply only to WPC extrusion, a fundamentally different manufacturing process from flat pressing; clearly, these models do not describe the FPWPC hot-pressing process. Considering all the specific features of FPWPC pressing, the development of a mathematical model of pressing will allow for the simulation of this process, providing a tool for the optimisation, control, and monitoring of the hot pressing of WPCs.

## 2. Development of the Mathematical Model

**Figure 1:**
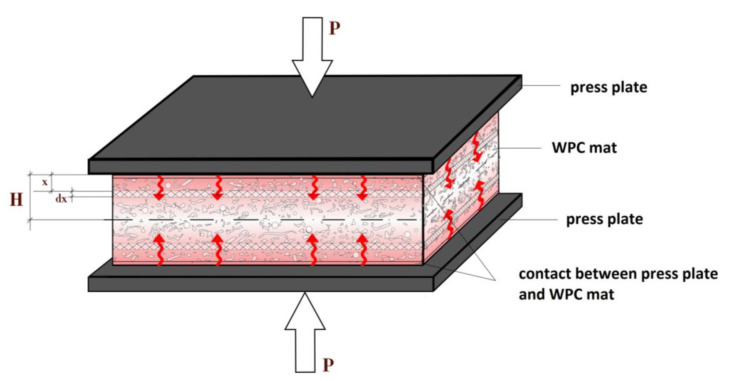
Hot-pressing process for making FPWPCs.

The time required to close and open the press plates, and the time required to increase and decrease the pressure, are determined by the type of flat-pressing equipment and any modifications to it. Primarily, the pressing time for FPWPC panels is determined by the time it takes to heat the mat through to the core layer (*H*) (Figure 1).

Fourier’s law of heat conduction states that the rate of heat transfer (*dQ*) through a material is directly proportional to its thermal conductivity (*λ*), the temperature difference across the material (represented by the temperature gradient $\frac{dt}{dx}$), and the area of the surface through which the heat flows (*F*). This relationship can be expressed mathematically as follows:

(1) $$\frac{dQ}{d\tau }=-\lambda \cdot F\cdot \frac{dt}{dx}.$$

On the other hand, over a certain period of time (*dτ*), the material is heated to a specific depth (*dx*), and the amount of heat required to heat the material at this depth is determined by the following:

(2) $$\frac{dQ}{d\tau }=q_{i}\cdot F\cdot \frac{dx}{d\tau },$$

where *q_i_* is the specific heat consumption for heating a unit volume of the composite material.

Fourier’s law states that the rate of heat transfer through a material is directly proportional to its thermal conductivity, the temperature difference across the material, and the area of the surface through which the heat flows [22]. Over a specified time, the material is heated to a particular depth, consuming a certain amount of thermal energy. Equating these terms gives the following:

(3) $$\frac{\partial }{\partial x}\left(\lambda (T)\frac{\partial T}{\partial x}\right)=c(T)\cdot \rho \cdot \frac{\partial T}{\partial \tau },$$

During pressing, the surfaces of the FPWPC mat reach the temperature of the press plates almost instantaneously. The temperature of the middle layers gradually increases, tending towards the surface temperature. Then, the temperature interval required to heat-treat FPWPC can be specified as follows:

(4) $$\frac{\partial T}{\partial x}=\frac{\left(T_{x}-T_{k}\right)}{\partial x}\;\;\frac{\partial T}{\partial \tau }=\frac{\left(T_{press}-T_{k}\right)}{\partial \tau },$$

where *T_k_*, *T_x_*, and *T_press_* are the temperatures of the middle layers (which can be assumed to be the ambient temperature) and the outer layers of the composite material (which can be assumed to be the temperature of the press plates, *T_press_*), and the target temperature to which the FPWPC needs to be heated, °C, respectively.

Then, the initial conditions will be as follows:

(5) $${\left.T\right|}_{\tau =0}=T_{0}.$$

and the boundary conditions will be as follows:

(6) $${\left.T\right|}_{x=h}=T_{press};\;{\left.\frac{\partial T}{\partial x}\right|}_{x=0}=0$$

Note that for the numerical solution to the system of equations, constant values for the thermophysical properties of wood (specific heat capacity, thermal conductivity, and thermal diffusivity) must be assumed. These properties are considered to be invariant with respect to both spatial coordinates and time.

The thermal conductivity of an FPWPC depends on the volumetric proportions of its wood and polymer components and can be calculated using a specific formula:

(7) $$\lambda (T)=\lambda_{pol}(T)\cdot \left(1+\frac{\varphi_{wood}}{\frac{\left(1-\varphi_{wood}\right)}{3}+\frac{\lambda_{pol}(T)}{\lambda_{wood}(T)-\lambda_{pol}(T)}}\right),$$

where *λ_wood_* and *λ_pol_* are the thermal conductivities of wood and polymer, respectively; *φ_wood_* is the volumetric fraction of wood particles in the composite material, expressed as a fraction.

Therefore, the volumetric fraction of wood particles can be calculated in the following manner:

(8) $$\varphi_{wood}=\frac{V_{wood}}{V_{WPC}}\;\mathrm{or}\;\left(1-\frac{V_{pol}}{V_{WPC}}\right),$$

where *V_WPC_* is the volume of the FPWPC and *V_wood_* is the volume of wood particles within the FPWPC.

The thermal conductivities of the wood and the thermoplastic polymer are determined using experimental linear equations [23]:

(9) $$\begin{array}{l} \lambda_{pol}(T)=\left(0.429-0.99\cdot 10^{-3}\cdot T\right)\\ \lambda_{wood}(T)=\left(0.361+1.876\cdot 10^{-4}\cdot T\right) \end{array}$$

To obtain FPWPCs with the best physical and mechanical properties, the wood–polymer mixture must be heated to a temperature equal to or slightly above the melt temperature of the thermoplastic polymer (*t_melt_*):

(10) $$T_{heat}=t_{melt}+10$$

The specific heat capacity can be determined by considering the heat required to heat each component of the composition and its mass fraction:

(11) $$Q_{n}=Q_{n.wood}^{W}+Q_{n.pol}+Q_{n.air}$$

In this case, the specific heat capacity of the FPWPC can be calculated using the following equation:

(12) $$c(T)=\frac{Q_{n.wood}^{W}+Q_{n.pol}+Q_{n.air}}{m_{WPC}\cdot (t_{melt}-T_{x}+10)}$$

Due to the low air content of FPWPCs, the heat capacity of the moist wood and thermoplastic polymer can be calculated by the following:

(13) $$\begin{array}{l} Q_{n.wood}^{W}=m_{wood}^{W}\cdot c_{wood}^{W}(T,W)\cdot (t_{melt}-T_{ê}+10)\\ Q_{n.pol}=m_{pol}\cdot c_{pol}(T)\cdot (t_{melt}-T_{ê}+10) \end{array},$$

where *m^W^_wood_* and *m_pol_* are the weight of the moist wood particles and the thermoplastic polymer in the FPWPC, respectively; *c^W^_wood_* and *c_pol_* are the specific heat capacities of the moist wood particles and the thermoplastic polymer, respectively; and *W* is the moisture content of the wood particles.

Then, the specific heat capacity of the moist wood particles, considering their moisture content (*W*) and temperature (*T*), can be determined as follows [24]:

(14) $$c_{wood}^{W}(T,W)=\frac{1131+4.19\cdot T+4190\cdot W}{(1+W)\cdot 1000}$$

The specific heat capacity of a thermoplastic polymer is temperature (*T*)-dependent and can be calculated [25] by the following:

(15) $$c_{pol}(T)=\frac{2550+3.43\cdot (T-130)}{1000}$$

We chose the finite element method as the numerical method to solve the boundary value problem (1)–(4). This method is based on the idea of approximating a continuous function with a discrete model built on a set of piecewise continuous functions defined on a finite number of sub-domains called finite elements. The investigated geometric domain is divided into elements in such a way that on each of them, the unknown function is approximated by a so-called trial function, which must adhere to both continuity requirements and the problem’s prescribed boundary conditions.

To implement the developed model, we used the mathematical package Matlab R2021b (9.11) (MathWorks, Natick, MA 01760-2098, USA), which has an extension package to incorporate the finite element method, Matlab PDE Toolbox (PDE). The functions “pdeinit” and “pdetool” launch the GUI application PDETool, which allows for interactive operations in preparing the PDE model (creating the geometry of the body, setting boundary conditions, the type of equation and its coefficients, constructing a computational mesh, etc.), obtaining a solution and visualising it.

To account for varying press plate temperatures, wood content, and FPWPC densities, we created a Matlab function “calculate.m” that numerically simulates the system and returns a matrix of nodal temperatures over time.

## 3. Materials and Methods

To validate our mathematical model of the heating process of FPWPC, we conducted experiments using wood particles with a 3% moisture content and recycled high-density polyethylene (rHDPE) waste (LLC Morozovych, Lviv, Ukraine). To establish the temperature–duration dependence of the FPWPC heating, we used thermocouples connected to an RT 0102 temperature controller (PJSC “Thermoprylad”, Lviv, Ukraine). The graphical dependencies were displayed on a personal computer monitor using the “rt0102” programme (PJSC “Thermoprylad”, Lviv, Ukraine). The FPWPC panels were pressed at a pressure of 3.5 MPa in a hot hydraulic press “xoMκo” (LLC “ODEK” Ukraine, Orzhiv, Ukraine) equipped with distance strips and automatic temperature control. The thickness of the FPWPC panels was 16 mm. The FPWPC mat was formed in an open press mould with a thermocouple placed in the middle layers. The experimental conditions are presented in Table 1.

## 4. Results

### 4.1. Mathematical Model

**Figure 2:**
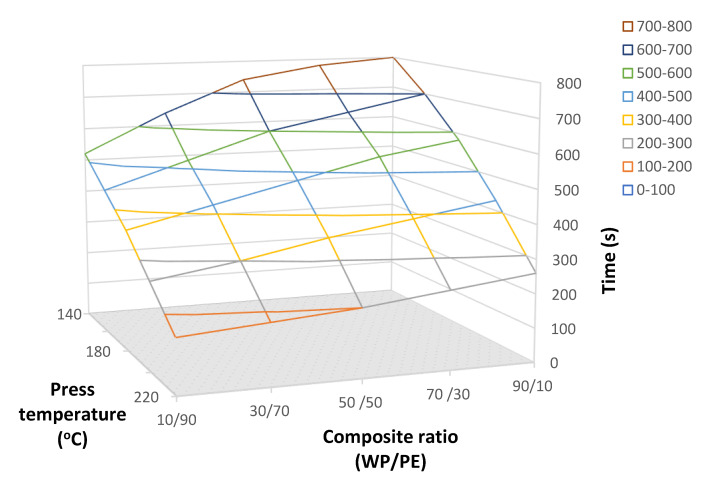
Mathematical modelling of the heating process of FPWPCs depends on press temperature, time of pressing, and composition ratio (wood particles/thermoplastic polymer).

The mathematical model we developed made it possible to plot the dependence of the heating time of the middle layers of an FPWPC on the press temperature and the composition ratio (Figure 2).

We established that the heating process of the FPWPCs could be divided into several stages. In the first stage, over 20–30 s, the core temperature of the mat remained virtually unchanged. This was due to the heat transfer from the press plates to the core layer of the mat. Notably, for FPWPCs with a higher wood-particle content, this process took longer than for materials with a higher thermoplastic-polymer content. This is related to the different thermal conductivities of wood and thermoplastic polymer, as confirmed by Equation (9). Moreover, an increase in FPWPC density led to the faster heating of the package due to closer contact between the mat elements.

In the second stage, we observed a nearly linear increase in the core layer temperature (almost up to 100 °C) as the heating continued, approaching the pressing temperature. This linearity is characteristic of materials with a high content of thermoplastic polymer, as well as of high pressing temperatures. An increase in the wood-particle content resulted in a decrease in the FPWPC mat temperature during the second stage and the subsequent transition to the third heating stage.

In the third stage, we recorded a substantial deceleration in the rate of temperature increase within the core layer of the FPWPC mat, particularly for materials with a 90% wood content and a minimum press plate temperature of 140 °C. Conversely, FPWPCs with a high thermoplastic-polymer content showed more linear heating to 120 °C after the first stage. Along with the compositional ratio, the pressing temperature exerted a significant influence on the heating dynamics of the FPWPCs. The relatively small temperature differential between the target and lower limit temperatures (only 20 °C) prolonged the heating process. Consequently, we concluded that this temperature regime was not suitable for the industrial-scale production of FPWPCs.

Similar dependencies have been observed by other researchers [7,12,18,24] studying conventional wood composite materials. We found that there was no increase in temperature inside the mat during the first 30 s of pressing. After this period, the temperature inside the mat increased rapidly until it reached 100 °C, after which the rate of increase began to slow dramatically. We suggest this rapid temperature increase in the interval from 30 to 110 s to be largely due to the movement of evaporated moisture from the area near the press plates towards the middle layers, as well as to the thermal conductivity of the wet wood particles. Once the temperature exceeded 100 °C, heat transfer by conduction became more significant, and further increases in temperature led to more evaporation of moisture from the core [18]. This effect was more pronounced in the FPWPC panels with a higher wood-particle content. The panels with more thermoplastic polymer exhibited a smoother transition.

### 4.2. Experiment

**Figure 3:**
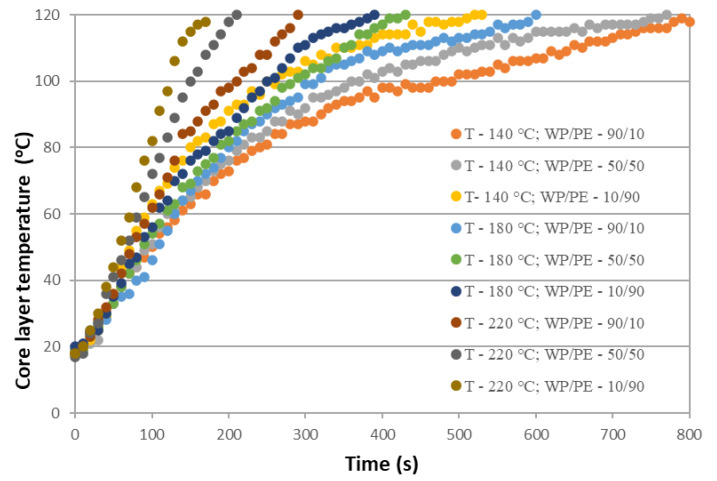
The effect of press temperature, time of pressing, and component ratio (wood particles/thermoplastic polymer) on the core layer temperature.

We displayed the experimental results graphically to illustrate the time of heating of the FPWPC as a function of press temperature and composite ratio (Figure 3).

The experimental results indicate that the heating time of the FPWPCs was directly proportional to the press temperature, which is connected to the dependence of thermal conductivity on the press temperature. However, the dependence of this parameter on the wood-particle content is more complex. The temperature rose rapidly to 100 °C at a wood-particle content of 90/10, but then the rate of heating decreased markedly. The presence of minor moisture content in the wood particles can account for this observation.

The thermal conductivity of water is a lot greater than that of both wood and polyethylene (by 4.0 and 2.5 times, respectively). Therefore, the heating of the FPWPC mat to a core temperature of 100 °C occurred rapidly even when it had a 90% wood-particle content. Additionally, the specific heat of the FPWPC mat increased with increasing moisture content, and this dependence exhibited a power–law effect. Nevertheless, the moisture content in the FPWPC is low because moisture is provided only by the wood particles, unlike other wood composite materials that use liquid thermosetting adhesives.

The heating process of the mat proceeded slowly after reaching a core temperature of 100 °C, which is explained by the low thermal conductivity of dry wood. The thermal conductivity of polyethylene is 1.6 times greater than that of wood, which would explain the faster heating of the mat due to the higher content of thermoplastic polymer in it. So, the heating of FPWPC panels with a high content of wood particles can be characterised by two distinct phases: (1) initial heating due to moisture evaporation; and (2) the subsequent heating of the dry wood particles. We found the heating rate to be more linear for the panels with lower wood content, and attribute this to the lower moisture content and the dominant thermal properties of the thermoplastic polymer.

### 4.3. Validation of the Mathematical Model

**Figure 4:**
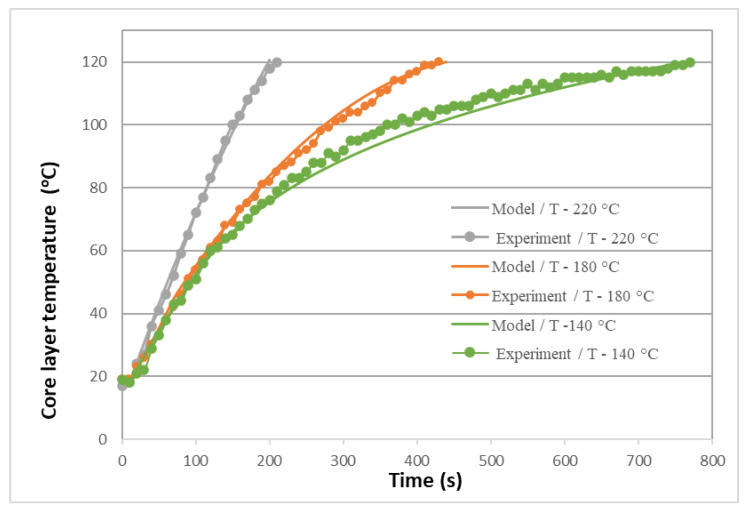
Verification of the mathematical model of heating FPWPCs: model and experimental data at different pressing temperatures.

**Figure 5:**
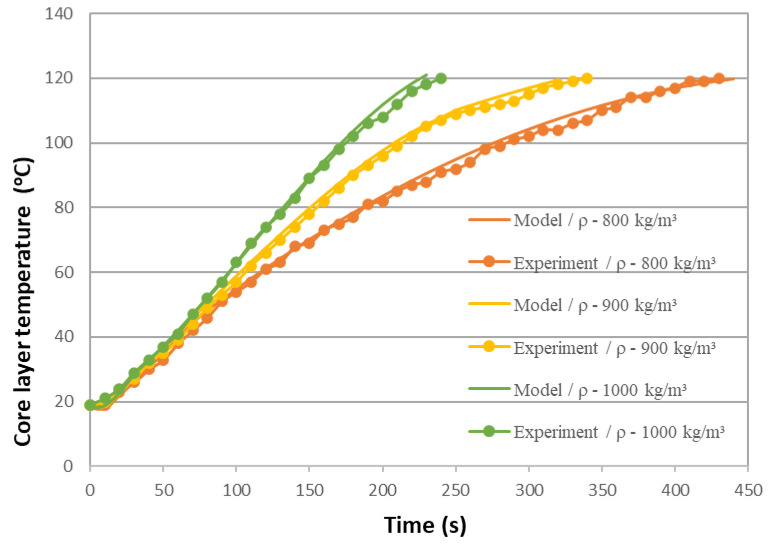
Verification of the mathematical model of heating FPWPCs: model and experimental data with different FPWPC densities.

A validation of the mathematical model is presented in Figure 4, which compares the predicted results with the experimental data. The heating rate of FPWPCs is shown to be directly proportional to their density, as illustrated in Figure 5.

There is a direct correlation between the mat heating time and the inverse of the predicted density of the FPWPC panels. This can be attributed to the direct proportionality between thermal conductivity and density, whereby denser materials facilitate more rapid heat transfer [24].

Other researchers [19] have found a similar dependence between the heating time of flat-pressed WPCs and the pressing temperature when the wood flour content was 50%. The hot-pressing process was terminated after 1300 s at a pressing temperature of 170 °C, 900 s at 190 °C, and 800 s at 210 °C. This roughly corresponded to the time needed to reach the target temperature of 160 °C in the core layer.

The verification results we obtained confirm the high accuracy and reliability of our mathematical model. The almost complete coincidence of the experimental data and modelling results (MAPE = 2.5%) allows us to use this model confidently for further research and prediction.

## 5. Conclusions

We can draw the following conclusions from our results: to accelerate the heating of FPWPCs, it is advisable to use high temperatures, which increases the productivity of the pressing equipment; the use of panels with a significant wood-particle content is impractical, as it significantly increases the time needed to press the panels; and it is recommended to use wood particles with a high moisture content, as this significantly reduces the heating time. The mathematical model we developed has a high degree of accuracy and can be used for further calculations of the pressing time of FPWPCs with variable parameters: press temperature, composite ratio, and the thickness and density of the mat. The model facilitates the optimisation of the hot-pressing process for flat-pressed WPCs at an industrial scale, enabling the precise calculations of pressing parameters across various temperatures and wood particle/thermoplastic polymer composition ratios. Furthermore, this model can be adapted to optimise the hot pressing of other thermoplastic polymers by substituting the thermal conductivity and specific heat capacities with those of the desired materials. Additionally, it supports the development of new composites and technologies for their production while enhancing our understanding of the complex interactions occurring during the hot-pressing process.

## Figures and Tables

**Table 1:** Experimental conditions.

Conditions	Symbol	Values
Press temperature (°C)	T	140, 180, 220
Composite ratio (wood particles/rHDPE)	WP/PE	10/90, 50/50, 90/10
Density of FPWPC (kg/m^3^)	*ρ*	800, 900, 1000

## Data Availability

The data that support the findings of this study are available upon reasonable request from the authors. Data are contained within the article.
